# Association of Quadratus Lumborum Muscle Stiffness with Chronic Low Back Pain Features: An Observational Study

**DOI:** 10.3390/medicina61020270

**Published:** 2025-02-05

**Authors:** Mónica López-Redondo, Davinia Vicente-Campos, Javier Álvarez-González, Alberto Roldán-Ruiz, Sandra Sánchez-Jorge, Jorge Buffet-García, Gabriel Rabanal-Rodríguez, Juan Antonio Valera-Calero

**Affiliations:** 1 Faculty of Health Sciences, Universidad Francisco de Vitoria, 28223 Madrid, Spain; davinia.vicente@ufv.es (D.V.-C.); j.alvarezglez.prof@ufv.es (J.Á.-G.); alberto.roldan@ufv.es (A.R.-R.); s.sjorge.prof@ufv.es (S.S.-J.); j.buffet.prof@ufv.es (J.B.-G.); 2 Department of Radiology, Rehabilitation and Physiotherapy, Faculty of Nursery, Physiotherapy and Podiatry, Complutense University of Madrid, 28040 Madrid, Spain; grabanal@ucm.es; 3 Grupo InPhysio, Instituto de Investigación Sanitaria del Hospital Clínico San Carlos (IdISSC), 28040 Madrid, Spain

**Keywords:** low back pain, quadratus lumborum, shear wave elastography, muscle stiffness

## Abstract

*Background and Objectives*: Low back pain (LBP) is highly prevalent and often associated with altered muscle function, including in the quadratus lumborum (QL) muscle. While some studies have highlighted the clinical relevance of QL muscle stiffness in LBP, the findings remain inconsistent, and the role of this parameter in relation to clinical severity indicators is not well understood. Considering the high prevalence of myofascial trigger points among patients, objectively and reliably quantifying QL stiffness and its association with other clinical parameters could improve the identification of early stages of the condition before other alterations become apparent. Therefore, this study aimed to explore the association between QL stiffness and multiple indicators of LBP severity. *Materials and Methods*: A cross-sectional observational study was conducted involving the participation of seventy-six patients suffering from chronic LBP. An ultrasound scanner with shear-wave elastography (SWE) was used to determine the participants’ QL stiffness. Additional information was collected on LBP-associated pain intensity, disability, central sensitization, and quality of life. *Results*: QL muscle stiffness was negatively correlated with pain intensity (*p* < 0.01) and central sensitization (*p* < 0.01), and it was positively correlated with physical quality of life (*p* < 0.01). Muscle stiffness influenced the variance in pain intensity along with physical quality of life, central sensitization, and chronicity (together explaining 49% of the variance) but did not explain the variance in central sensitization. *Conclusions*: Assessing QL muscle stiffness in patients with LBP is recommended, as greater muscle softness is linked to higher pain intensity, central sensitization, and poorer physical quality of life. Regression analyses further highlighted that QL stiffness helps explain the variance in pain intensity, physical quality of life, central sensitization, and chronicity, but it did not directly affect the central sensitization variance.

## 1. Introduction

The quadratus lumborum (QL) is a muscle anatomically found in the posterolateral aspect of the lumbar region, inserting in the iliac crest, the transverse processes of the lumbar vertebrae, and the lower border of the 12th rib [1]. Although its main functions are dynamic, comprising lumbar spine extension and lateral flexion, it is also considered an inspiratory accessory muscle [2].

From a clinical perspective, the QL muscle plays a key role in low back pain (LBP), as significant functional findings have reported. For instance, electromyographic studies demonstrated how patients suffering from recurrent LBP showed a redistribution of activity compared with asymptomatic individuals [3,4]. However, anatomical findings remain uncertain, since imaging studies reported controversial results [5]. For instance, Kamaz et al. [6] reported that chronic LBP causes atrophy of the QL muscle while Sions, after examining L2 to L5 levels, concluded that LBP did not interact with the QL cross-sectional area nor with the fatty infiltration percentage [7].

Considering that several studies reported a high prevalence of active myofascial trigger points (MTrPs) in patients with non-specific LBP [8,9,10,11,12], which are defined as “hyperirritable painful spots located in taut bands which elicits local and referred pain recognized by the patient if stimulated mechanically” [13], assessing muscle stiffness may provide clinically important information to identify early stages of diseases when morphological abnormalities are not evident in gray-scale images.

Sonoelastography is employed to gather information about tissue elasticity in this context, serving as a complementary tool to B-mode ultrasound imaging (US). Although various elastography methods for evaluating musculoskeletal structures are outlined in the literature [14], shear wave elastography (SWE) is considered the most valid method. SWE utilizes mechanical shear waves (generated by the compressive acoustic waves used in acquiring B-mode images) to provide information about the velocity of shear wave propagation (which is closely associated with tissue stiffness).

Given that current recommendations emphasize the assessment of muscle tenderness, particularly for the QL muscle and the gluteus medius in patients with LBP, and considering the poor reliability in determining the presence, number, and location of MTrPs [10], there is an increasing interest in exploring objective methods to quantify muscle stiffness and analyze its association with clinical severity indicators (including pain intensity, pain chronicity, recurrence, pain-related disability, central sensitization, and quality of life). Therefore, considering that SWE is a reliable tool for assessing QL stiffness, as demonstrated by Zhou et al. [15], the general aim of this study was to explore the association of general QL stiffness with indicators of chronic LBP severity. Additionally, this research aimed to conduct a regression model to discuss the individual contribution of muscle stiffness (if it contributed) to the variance of the clinical severity indicators. Given the theoretical framework outlined on the characteristics of MTrPs and their relationship with LBP, the authors hypothesized that the stiffness of the QL muscle assessed with SWE would show a significant association with clinical severity indicators of chronic LBP (i.e., increased QL stiffness may be correlated with greater pain intensity, increased central sensitization, and worse quality of life, and the correlation would be significant) and would explain a significant percentage of the variance in pain intensity, disability, central sensitization inventory scores, and quality of life.

## 2. Materials and Methods

### 2.1. Study Design

Between February and May 2024, a longitudinal observational study was conducted to determine the association among several clinical severity indicators (including QL muscle stiffness) in a physiotherapy clinic located in a private university located in Madrid (Francisco de Vitoria University). To enhance the quality of this report, the recommendations listed in the STROBE checklist [16] and the EQUATOR guidelines [17] were followed. Additionally, the rights of the participants were considered in accordance with the Declaration of Helsinki, and a Local Ethics Committee (ID: UFV 15/2024) provided oversight and approval for this study protocol prior to data collection.

### 2.2. Participants

A sample of patients suffering from chronic LBP (established by “the persistence of pain beyond 3 months of symptoms”) [18] were recruited by posting local announcements around the campus. Inclusion criteria included being aged between 18 and 65 years and reporting at least one clinically relevant episode of LBP within the last year (an episode severe enough to require medical attention due to the intensity or disability caused by the symptoms), without neurological signs, and reporting scores of at least 2 points out of 10 on the Visual Analogue Scale [19] and at least 12 points out of 100 on the Oswestry Disability Scale [20] at the moment of data collection to cover the widest spectrum of clinical presentations.

Subjects being under any pharmacological treatment or medical condition potentially affecting the muscle tone or having a history of spine surgeries, neuropathies (i.e., radiculopathies or myelopathies), serious medical conditions (e.g., tumors, fractures, neurological disorders, or any systemic disease), reporting any identifiable pathoanatomical cause explaining the symptoms (e.g., specific low back pain [21]) or clinically relevant asymmetries, or suffering widespread painful conditions such as fibromyalgia or osteoarthritis were systematically excluded from the study. Volunteers passing this first screening were asked to read, understand, and sign an informed written consent to be scheduled for the data collection.

### 2.3. Sample Size Calculation

The minimum sample size needed for studies examining multivariate correlations was determined using Harry’s formula [22] (*n* = 50 + number of variables), which has been proven to be a reliable rule with sufficient power for detecting associations and conducting factor analyses [23]. Given that we included 8 variables, the correlation matrix required at least 58 participants to meet the acceptable threshold (*n* = 50 + 8 = 58). Regarding the sample size calculation for the regression models, a range from 10 to 15 participants per potential predictor (limiting up to five predictors in order to avoid modelling overestimation) was considered appropriate [24]. Therefore, the regression analyses required a minimum sample size of 75. As this was a cross-sectional study with no follow-up period, participant attrition was not applicable.

### 2.4. Quadratus Lumborum Muscle Stiffness

One experienced physiotherapist examiner with more than 10 years of experience in musculoskeletal US and clinical experience in the management of patients with musculoskeletal conditions acquired all the images. The examiner was blinded to the clinical information of the participants to minimize potential biases. The US device used for collecting all the images was a Canon Aplio A device with a convex transducer 8C1, and the parameters for all the exams were standardized (frequency = 5 MHz, gain = 80 dB, dynamic range = 60, and depth = 12 cm).

Participants were positioned in a lateral decubitus posture, ensuring spinal and lower limb neutrality. For this purpose, a wedge-shaped cushion was strategically placed posterior to the upper thoracic region to maintain a perpendicular orientation of the torso relative to the examination table. Additionally, a square cushion was inserted between the thighs to maintain hip joint neutrality. If necessary, a towel was utilized to support and maintain a neutral lumbar spine alignment [15]. All participants were instructed to relax the muscles during the procedure in order to prevent morphological bias attributable to muscle contractions [25].

**Figure 1:**
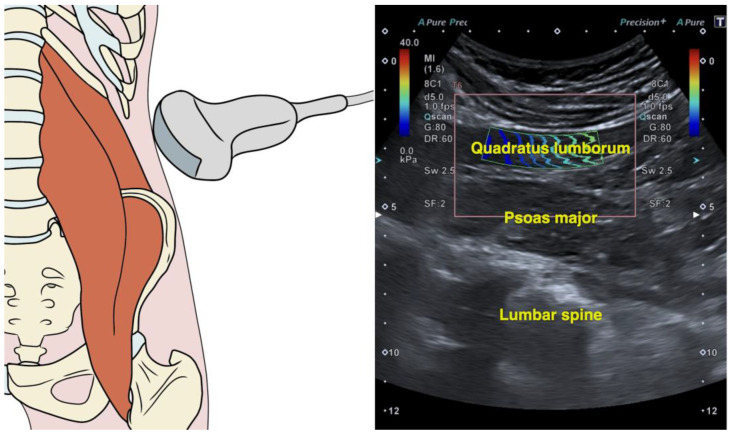
Illustration indicating the transducer placement, the corresponding shear wave elastography image, and the region of interest for measuring quadratus lumborum stiffness.

The transducer was initially positioned superior to the iliac crest along the mid-axillary line. Then, its cranial aspect was pivoted posteriorly by approximately 20° to achieve visualization of the L4 vertebra at the center of the imaging field (Figure 1). In this location, the quadratus lumborum was identified as the relatively hypoechoic muscle overlaying the psoas major muscle (visualized as the muscle overlying the vertebral bodies). Lastly, the region of interest was established on the center of the muscle, covering at least 50% of the muscle and excluding the fasciae, using a freely drawn quantification box (Figure 1). The side examined in patients reporting unilateral pain was the symptomatic one. In patients reporting bilateral pain, the mean average of both sides was calculated. The rationale behind this decision was that duplicating patients (by treating the left and right sides as separate measures) in the correlation analysis would introduce several methodological challenges and biases. First, it would violate the assumption of independence required for correlation analyses, as the left and right sides of the same patient are inherently related due to shared physiological, biomechanical, or pathological factors. This lack of independence could distort the results and artificially inflate the strength of observed correlations. Additionally, this approach would lead to an overrepresentation of individual data, as each patient would effectively contribute more than one data point to the analysis. This would create an imbalance, giving undue weight to patients with bilateral data compared to those with unilateral data, which would reduce the generalizability of the findings.

Moreover, treating both sides as separate measures could artificially reduce variability in the dataset, as the two sides of the same patient are likely to be more similar to each other than to other patients. This reduction in variability could result in an overestimation of the strength of associations, leading to misleading conclusions. Similarly, including both sides as independent data points would inflate the sample size without adding new information, potentially leading to false confidence in the statistical results and narrower confidence intervals that would not accurately reflect the true variability of the population.

### 2.5. Clinical Severity Indicators

A separate examiner was responsible for gathering data on clinical severity indicators. A clinical history form was completed, documenting the duration (in months) of the patient’s LBP and the number of LBP episodes experienced over the past year.

Pain intensity was measured using the Visual Analogue Scale (VAS), a widely recognized and valid tool for assessing pain levels. The VAS consists of a straight 10 cm line, with its endpoints representing the extremes of pain perception: 0 indicating no pain and 10 signifying the most severe pain imaginable. The patient’s score is determined by measuring the distance in centimeters from the “no pain” end to their marked point. The VAS score ranges between 0 and 10, with higher values indicating more severe pain [26].

Pain-related disability was evaluated using the Oswestry Disability Index (ODI), a reliable and validated questionnaire composed of 10 sections. Each section contains six statements rated on a scale from 0 to 5, where 0 represents no disability and 5 denotes the highest level of disability. The total score categorizes patients into one of five disability levels: minimal (0–20), moderate (21–40), severe (41–60), crippling (61–80), or total disability (81–100) [20].

The Central Sensitization Inventory (CSI), a self-reported questionnaire, was used to assess symptoms associated with central sensitization. This inventory includes 25 items, each rated on a 5-point Likert scale ranging from 0 (never) to 4 (always). The total score is the sum of all responses, with possible values between 0 and 100, where higher scores indicate a greater extent of central sensitization [27].

To assess patients’ quality of life, the SF-12 questionnaire was utilized. This tool comprises 12 questions covering eight health dimensions: physical functioning, role limitations due to physical health issues, bodily pain, general health perception, vitality, social functioning, role limitations due to emotional health problems, and mental well-being. Scores are derived using a specific algorithm that generates two summary measures—Physical Component Summary and Mental Component Summary—both ranging from 0 to 100, with higher values reflecting better health-related quality of life [28].

### 2.6. Statistical Analysis

All data were processed and analyzed using the Statistical Package for the Social Sciences (SPSS) version 29.1.1 (Armonk, NY, USA) for Mac OS. Statistical tests were two-tailed, with a significance threshold set at *p* < 0.05. Initially, histograms and Shapiro–Wilk tests were used to assess the distribution of continuous variables. Descriptive statistics were then applied to summarize the demographic and clinical characteristics of the sample. Given prior findings from Zhou et al. [15] on the impact of gender on muscle stiffness, comparisons between males and females were conducted using independent-samples Student’s T-tests, with results reported as mean differences, 95% confidence intervals, and *p*-values. Additionally, an analysis of left–right muscle stiffness differences was performed to confirm that no significant disparities existed (*p* > 0.05), ensuring the justification for calculating the average stiffness of both sides as a representative measure.

Pearson correlation coefficients (r) were used to evaluate the strength and direction of associations among the measured variables. Furthermore, r coefficients were analyzed to detect multicollinearity and shared variance (considered significant at r > 0.80) to mitigate potential bias and overestimation in the regression model calculations [29].

Following this, two multivariate linear stepwise regression models were constructed, one for pain intensity and another for central sensitization. Variables demonstrating the strongest correlations, without shared variance, and meeting statistical significance (*p* < 0.05) were sequentially incorporated into the forward stepwise regression model. The critical F-value threshold for significance was set at *p* < 0.05. Adjusted R² values were documented at each stage to quantify the variance contribution of each included variable [30].

## 3. Results

During the recruitment period, 80 individuals expressed interest in participating in this study. Four participants were excluded due to neuropathic complaints. The remaining 76 participants (*n* = 37 females) were found to be valid and analyzed. Table 1 summarizes the demographic and clinical characteristics of the patients included in this study, comparing males and females. The analysis of demographic characteristics revealed that there were no significant differences in age nor BMI between females and males (both *p* > 0.05). However, males were significantly heavier and taller than women (both *p* < 0.001). Regarding clinical characteristics, females reported a significantly higher number of recurrences of low back pain episodes in the past year compared to males (*p* < 0.001). There were no significant differences in the chronicity of low back pain, pain intensity, or quality of life between genders (all *p* > 0.05). However, pain-related disability (*p* = 0.003) and central sensitization (*p* < 0.001) scores were significantly higher for females compared to males. Finally, there was no significant difference in quadratus lumborum stiffness between females and males (*p* > 0.05).

The multivariate correlation matrix is reported in Table 2. Muscle stiffness was negatively correlated with pain intensity (*p* < 0.01) and central sensitization (*p* < 0.01) and positively correlated with physical quality of life (*p* < 0.01). In addition, pain intensity was positively correlated with the recurrence of episodes (*p* < 0.05) and central sensitization (*p* < 0.01), pain-related disability was positively correlated with recurrence (*p* < 0.05) and central sensitization (*p* < 0.05), central sensitization was negatively correlated with both physical and mental quality of life (both *p* < 0.01), physical quality of life was negatively correlated with pain intensity (*p* < 0.01), disability (*p* < 0.01), and central sensitization (*p* < 0.01), and mental quality of life was negatively correlated with central sensitization (*p* < 0.01).

The regression models are detailed in Table 3. This research calculated the two models showing the greatest explained variance (pain intensity and central sensitization). The regression analysis for pain intensity included four steps. In Step 1, physical quality of life explained 32.6% of the variance. In Step 2, adding central sensitization increased the explained variance to 39.9% (with an individual contribution of 7.3%). Step 3 included quadratus lumborum stiffness, further increasing the explained variance to 43.9% (with an additional 4.0%). Step 4 added chronicity, explaining 49.1% of the variance (with an additional 5.2%). On the other hand, the regression analysis for central sensitization started with pain intensity in Step 1, explaining 25.9% of the variance. Step 2 added physical quality of life, increasing the explained variance to 29.1% (with an additional 3.2%). Step 3 included recurrence, further increasing the explained variance to 34.2% (with an additional 5.1%).

## 4. Discussion

This is the first study assessing if the evaluation of QL muscle stiffness with SWE is clinically relevant in patients suffering from LBP. The most relevant findings of the study were that QL muscle stiffness was significantly associated with several key clinical severity indicators. Notably, lower muscle stiffness was correlated with greater pain intensity, greater central sensitization, and worse physical quality of life, suggesting that increased stiffness might be a marker of better clinical outcomes in certain aspects. Additionally, the regression models revealed that muscle stiffness played a relevant role in pain intensity, as it partially explained its variance. Physical quality of life, central sensitization, QL stiffness, and chronicity collectively explained nearly half of the variance in pain intensity. In terms of central sensitization, QL muscle stiffness did not contribute. However, pain intensity, physical quality of life, and recurrence explained a substantial portion of the variance.

Zhou et al. [15] analyzed the impact of gender and physical activity on muscle stiffness, describing how asymptomatic females and physically inactive individuals exhibited higher QL stiffness. The authors explained that the gender differences in muscle stiffness could be attributed to variations in muscle composition, with females typically having a higher fat content and different muscle fiber types compared to males. In contrast, our results not only revealed comparable stiffness in a sample of patients suffering chronic LBP but also softer QL stiffness scores compared to the asymptomatic sample studied by Zhou et al. [15] and a negative association with the clinical severity indicators. Although further analyses are needed to corroborate the hypothesis, the muscle composition variations and altered function attributed to LBP described in previous studies may explain the discrepancy (as these histological differences cannot be attributed exclusively to gender differences).

Although some authors reported that chronic LBP causes QL (and other muscles including lumbar multifidus, psoas, and paraspinal muscles) atrophy [31,32], the evidence is generally controversial [5,7,33,34]. However, non-histological factors such as muscle recruitment at rest or during functional tasks also have a critical impact on muscle stiffness [3,4,35]. Colloca and Hinrichs [36] have provided insight into how disruptions in the neuromuscular balance are associated with LBP. They discussed the flexion–relaxation phenomenon, which found myoelectric silence consistent with increased load sharing of the posterior discoligamentous passive structures during trunk flexion. The phenomenon indicates that during flexion, the load is shifted to the passive structures of the spine (such as ligaments and intervertebral discs), leading to LBP pain and disability. While the lumbar erector spinae are persistently activated among patients with LBP (a finding that may represent the body’s attempt to stabilize injured or diseased spinal structures via reflexogenic ligamento–muscular activation, thereby protecting them from further injury and avoiding pain), there is a myoelectric silencing of the QL. This background provides a comprehensive rationale to explain the association between a softer QL and worse clinical presentations and the reduced stiffness of patients with LBP in comparison with asymptomatic subjects.

The controversy between the high prevalence of an MTrP (which is defined as a “palpable nodule in a taut band” [37]) and the decreased stiffness found in this study should also be discussed, as greater stiffness could be expected based on this definition. First, there is a clear flaw in the diagnosis of MTrPs, because in the absence of objective tools to demonstrate this stiffness, it depends on a physical examination and the pain responses reported by the patients [38]. In previous studies conducted on the cervical region, the authors found a clear inconsistency between the subjective stiffness perceived by the patients and the objective stiffness metrics obtained using SWE [39]. Dieterich et al. [39] compared muscle stiffness using SWE at five different cervical sites between women with chronic non-specific neck pain and asymptomatic women. The results showed that the patients did not exhibit higher objective muscle stiffness than the asymptomatic women across various muscle-specific regions and tasks, despite their subjective perception of increased stiffness. Similarly, Wolff et al. [40] found that patients with idiopathic chronic neck pain had a softer sternocleidomastoid muscle compared to asymptomatic controls during forward reaches. The authors also attributed the results to pain-avoidance movement strategy adaptations, as contrasting findings were observed for the upper trapezius muscle, and the stiffness changes were independent of muscle activity [40].

In addition, another study [41] investigated the stiffness properties of active and latent MTrPs located in the upper trapezius muscle, compared the properties with pain-free control locations, and examined the relationship between muscle stiffness and clinical severity outcomes. The study revealed no significant differences among active, latent, or control regions. Additionally, there were no correlations found between the SWE metrics and pain intensity, pain extent, pain-related disability (similar to the findings of Xie et al. [42]), or pressure pain thresholds. These findings were reinforced in a clinical trial revealing that specific interventions improving pain intensity and pain-related disability had no effects on muscle stiffness [43].

### 4.1. Practical Implications

The findings of this study provide several important practical implications for the clinical management of patients with LBP. First, the significant association between QL muscle stiffness and key clinical severity indicators highlights the potential of SWE as a valuable tool for evaluating muscle stiffness in this patient population. The inverse relationship between QL stiffness and pain intensity, central sensitization, and physical quality of life suggests that increased QL stiffness may serve as a marker of more favorable clinical outcomes in certain aspects of LBP, challenging the traditional belief that greater stiffness is associated with worse clinical presentations.

From a clinical perspective, these findings emphasize the relevance of integrating an SWE-based assessment of QL muscle stiffness into routine diagnostic and monitoring protocols for patients with LBP. By identifying patients with lower QL stiffness, clinicians may be better equipped to stratify individuals at a higher risk for worse pain intensity and an impaired quality of life, allowing for tailored therapeutic interventions. Furthermore, the significant role of QL stiffness in explaining the variance in pain intensity underscores its potential utility in personalized treatment planning, particularly in targeting pain management strategies.

The regression models also provide critical insights into the multifactorial nature of LBP. The combination of physical quality of life, central sensitization, QL stiffness, and chronicity explaining nearly half of the variance in pain intensity underscores the importance of adopting a multimodal approach in managing LBP. This highlights the need for interventions that not only address musculoskeletal factors, such as muscle stiffness, but also consider the broader biopsychosocial determinants of pain.

Importantly, the lack of a direct contribution of QL stiffness to central sensitization suggests that a stiffness assessment alone may not fully capture the complex neural mechanisms underlying central sensitization. This reinforces the need for comprehensive clinical evaluations that include assessments of pain intensity, recurrence, and physical quality of life to understand and address central sensitization in LBP management.

In terms of rehabilitation strategies, the observed association between QL stiffness and LBP severity aligns with the evidence supporting the effectiveness of adapted physical exercise and multidisciplinary approaches. Baroncini et al. [44] demonstrated that adapted physical exercise and multidisciplinary strategies are among the most effective approaches for reducing pain and disability in patients with chronic LBP. Adapted physical exercise, which includes individualized regimens tailored to the patient’s needs and functional limitations, promotes strength, flexibility, and balance while addressing kinesiophobia and psychosocial factors. Similarly, multidisciplinary approaches, which combine physiotherapy, psychological support, and education, have been shown to enhance functional recovery and reduce disability. Given these findings, incorporating tailored exercise programs and multidisciplinary interventions into the treatment plans for patients with LBP could maximize their therapeutic outcomes. These strategies not only address QL stiffness but also improve the broader functional and psychological aspects of chronic pain. Importantly, such approaches could help clinicians move beyond stiffness as a single focus and adopt comprehensive management strategies that optimize patient care and outcomes.

### 4.2. Limitations

Despite the significant findings of this study, several limitations should be acknowledged. First, even if the minimum sample size to obtain statistically acceptable power was reached, the sample was not heterogeneous enough to provide definitive conclusions. Further research including participants with greater clinical severity scores (especially pain-related disability and central sensitization) and side-specific analyses are needed to confirm the generalizability of the results. Second, although the SWE protocol demonstrated excellent reliability, it was tested in asymptomatic subjects. Specific reliability analyses in patients suffering from LBP are needed, as the minimum detectable changes and accuracy of measurements might be different due to the histological and functional differences reported in the literature between cases with LBP and asymptomatic subjects. In addition, further research should specifically assess the impact of other variables (such as psychological stress, physical activity levels, and lifestyle habits), as these could be potential cofounding factors which might have influenced the results. Finally, LBP is inherently multifactorial (e.g., individual, psychological, biomechanical, nociceptive, behavioral, contextual, social, and occupational factors), and further research considering the etiology of LBP is needed to confirm if these findings are consistent.

## 5. Conclusions

This study highlights the importance of assessing the stiffness of the QL muscle in patients with chronic LBP, as the results suggested that lower muscle stiffness is associated with greater pain intensity and central sensitization, along with worse physical quality of life scores. Therefore, QL stiffness should be considered in patients with LBP, as increased QL stiffness might be associated with better clinical outcomes in specific aspects of chronic LBP. In addition, the regression analyses highlighted the role of QL muscle stiffness in explaining the variance of pain intensity, alongside physical quality of life, central sensitization, and chronicity. Although QL stiffness did not contribute to central sensitization variance, pain intensity, physical quality of life, and recurrence collectively, it explained a substantial portion of the variance in central sensitization.

## Figures and Tables

**Table 1:** Descriptive analyses of demographic and clinical characteristics of the sample.

Variables	Subjects with Low Back Pain (*n* = 76)		Difference (95% Confidence Interval)
	Females (*n* = 37)	Males (*n* = 39)	
**Demographics**			
Age, years	30.8 ± 13.7	26.1 ± 8.6	4.6 (−2.9; 12.1) *p* = 0.221
Weight, kg	66.6 ± 15.0	81.9 ± 12.6	−15.3 (−24.0; −6.6) *p* < 0.001
Height, m	1.65 ± 0.04	1.76 ± 0.08	−0.11 (−0.15; −0.08) *p* < 0.001
BMI, kg/m^2^	24.8 ± 5.7	26.5 ± 2.7	−1.6 (−4.6; 1.4) *p* = 0.287
**Clinical Characteristics**			
Recurrence (episodes last year, n)	12.1 ± 1.4	9.6 ± 1.3	2.5 (1.89; 3.11) *p* < 0.001
Chronicity (months, n)	65.8 ± 50.0	58.9 ± 46.3	6.9 (−14.7; 28.5) *p* = 0.525
Pain Intensity (VAS, 0–10)	4.9 ± 1.5	4.7 ± 1.4	0.2 (−0.4; 0.8) *p* = 0.522
Related Disability (ODI, 0–100)	13.4 ± 7.1	9.0 ± 4.9	4.4 (1.6; 7.3) *p* = 0.003
Central Sensitization (CSI, 0–100)	42.6 ± 13.3	29.3 ± 12.6	13.3 (7.5; 19.1) *p* < 0.001
Physical Quality of Life (SF-12, 0–100)	45.4 ± 10.0	47.0 ± 8.9	−1.60 (−5.9; 2.7) *p* = 0.460
Mental Quality of Life (SF-12, 0–100)	42.1 ± 10.3	46.3 ± 11.3	−4.2 (−8.9; 0.5) *p* = 0.081
Quadratus Lumborum Stiffness (SWS, m/s)	2.41 ± 0.65	2.47 ± 0.54	−0.06 (−0.33; 0.21) *p* = 0.669

Abbreviations: CSI: Central Sensitization Inventory; ODI: Oswestry Disability Scale; SWS: shear wave speed; VAS: Visual Analogue Scale.

**Table 2:** Correlation matrix analyzing the associations among clinical severity indicators and quadratus lumborum muscle stiffness.

	1	2	3	4	5	6	7
1. Duration (months)							
2. Recurrence (episodes)	n.s.						
3. Pain Intensity (VAS)	−0.372 **	0.231 *					
4. Disability (ODI)	n.s.	0.290 *	n.s.				
5. Central Sensitization (CSI)	n.s.	0.483 **	0.576 **	0.283 *			
6. Physical Quality of Life (SF-12)	n.s.	n.s.	−0.650 **	−0.570 **	−0.536 **		
7. Psychological Quality of Life (SF-12)	n.s.	n.s.	n.s.	n.s.	−0.470 **	n.s.	
8. Muscle Stiffness (SWS)	n.s.	−0.260 *	−0.507 **	n.s.	−0.441 **	0.403 **	n.s.

* *p* < 0.05; ** *p* < 0.01. Abbreviations: CSI: Central Sensitization Inventory; ODI: Oswestry Disability Scale; SWS: shear wave speed; VAS: Visual Analogue Scale.

**Table 3:** Summary of the regression analyses to determine contributors of low back pain intensity and central sensitization.

	Predictor Outcome	R^2^ Adj	B	SE B	95% CI	β	t	P
Pain Intensity	Step 1 Physical Quality of Life	0.326	−0.085	0.013	−0.111, −0.059	−0.578	−6.486	<0.001
	Step 2 Physical Quality of Life CSI	0.399	−0.063 0.031	0.014 0.009	−0.091, −0.036 0.013, 0.049	−0.431 0.319	−4.545 3.362	<0.001 0.001
	Step 3 Physical Quality of Life CSI QL Stiffness	0.439	−0.053 0.027 −0.542	0.014 0.009 0.206	−0.081, −0.025 0.009, 0.045 −0.952, −0.131	−0.361 0.28 −0.233	−3.785 3.026 −2.625	<0.001 0.003 0.010
	Step 4 Physical Quality of Life CSI QL Stiffness Chronicity	0.491	−0.053 0.029 −0.381 −0.007	0.013 0.009 0.204 0.002	−0.080, −0.027 0.012, 0.046 −0.786, 0.024 −0.012, −0.002	−0.363 0.296 −0.164 −0.245	−3.994 3.345 −1.872 −3.044	<0.001 0.001 0.048 0.003
Central Sensitization Inventory	Step 1 Pain Intensity	0.259	5.299	0.957	3.397, 7.201	0.517	5.539	<0.001
	Step 2 Pain Intensity Physical Quality of Life	0.291	3.855 −0.368	1.147 0.169	1.574, 6.135 −0.704, −0.032	0.376 −0.244	3.362 −2.181	0.001 0.032
	Step 3 Pain Intensity Physical Quality of Life Recurrence	0.342	3.000 −0.433 0.027	1.147 0.164 0.010	0.717, 5.282 −0.760, −0.106 0.007, 0.047	0.293 −0.287 0.250	2.614 −2.635 2.738	0.011 0.010 0.008

## Data Availability

The raw data supporting the conclusions of this article will be made available by the authors on request.

## Abbreviations

The following abbreviations are used in this manuscript:

CSI	Central Sensitization Inventory
LBP	Low back pain
MTrP	Myofascial Trigger Point
ODI	Oswestry Disability Index
QL	Quadratus lumborum
STROBE	Strengthening the reporting of observational studies in epidemiology
SWE	Shear wave elastography
VAS	Visual Analogue Scale
